# Military affected by the first wave of COVID-19 in Senegal: stress and resilience factors during care

**DOI:** 10.11604/pamj.2024.47.53.36263

**Published:** 2024-02-08

**Authors:** Serigne Modou Ndiaye, Diambéré Séga Dembélé, Moustapha Lo, Adama Fané, Florentine Mbengue Diagne, Khadidiatou Konaré Dembélé, Khadim Fall, Mbayang Ndiaye Djiba, Sokhna Ndiaye, Tabara Sylla Diallo

**Affiliations:** 1 Psychiatric Service, Army Training Hospital, Hôpital Principal de Dakar, Dakar, Senegal,; 2 Psychiatric Service, Ouakam Military Hospital, Dakar, Senegal,; 3 Dialogue Montreal, Quebec, Canada

**Keywords:** COVID-19, stressors, resilience, military, Senegal

## Abstract

COVID-19 had a psychological impact on the population, particularly those affected. Our objective was to investigate stress and resilience factors in the Senegalese soldiers affected during the first wave of COVID-19. Our retrospective and qualitative study included military personnel listed as contacts, suspects, or positive cases and supported by the Armed Forces Psychological Support Program during the period of isolation. The stress factors were health-related, sociological, and occupational. The conditions and the experience of isolation, stigmatization, and suspension of their professional projects were concerns for the soldiers. They had relied on personal, familial, and professional resources to cultivate resilience during the quarantine. Isolation during the pandemic showed psychological consequences, the foundations of which have been found in our study.

## Introduction

The coronavirus disease (COVID-19) and the mechanisms put in place to control it have had a psychological impact on the population, especially those affected [1]. In Senegal, the Ministry of Health and Social Action (MHSA), through the National Committee for Epidemic Management (NCEM), has gradually defined and adapted the case management policy during the first wave of the pandemic [2]. The NCEM took into consideration the social and psychological consequences through the psychosocial commission. This disease has not spared Senegal's defense and security forces who had to undergo the measures of care in force at the national level. They had an additional psychological follow-up from the Armed Forces Psychological Support Program (AFPSP), reflecting and taking into account their specificities. The purpose of our study was to investigate psychological issues in soldiers affected by COVID-19, particularly stress and resilience factors.

## Methods

Our study included Senegalese military personnel affected by the first wave of COVID-19. They were active military, deployed on the national territory or in operation outside the country, and attended by the medical structures. They all met the definition of contact, suspected or positive cases according to the Standard Operating Procedure (SOP 01) of the procedures manual for the COVID-19 outbreak response [2], established by the Health Emergency Operations Center (HEOC). We considered those who received additional psychological support from the AFPSP. This support was carried out at the request of their unit medical doctor, the medical authorities of the Armed Forces Health Service Directorate, or as a result of media coverage of the situation of those involved. The study did not include service members who couldn´t be reached by telephone or who declined psychological support.

Our study was retrospective, and qualitative, covering the period from April 12^th^ to July 20^th^, 2020, from the first to the last request for psychological support and during the first wave of the pandemic in Senegal. The study was based on data collected during individual psychological support telephone interviews, during the care period. They were made based on the methodology recommended by the psychosocial commission of the NCEM, which was responsible for support at the national level. Interview notes have made it possible to identify medico-administrative data on military status, COVID-19 status, isolation sites, stress, and resilience factors during the isolation period. Data on stress and resilience factors were coded and categorized into health, sociological, and work-related factors using MAXQDA 2020 software. Our study respected the ethical principles of the Helsinki Declaration.

## Results

**Figure 1:**
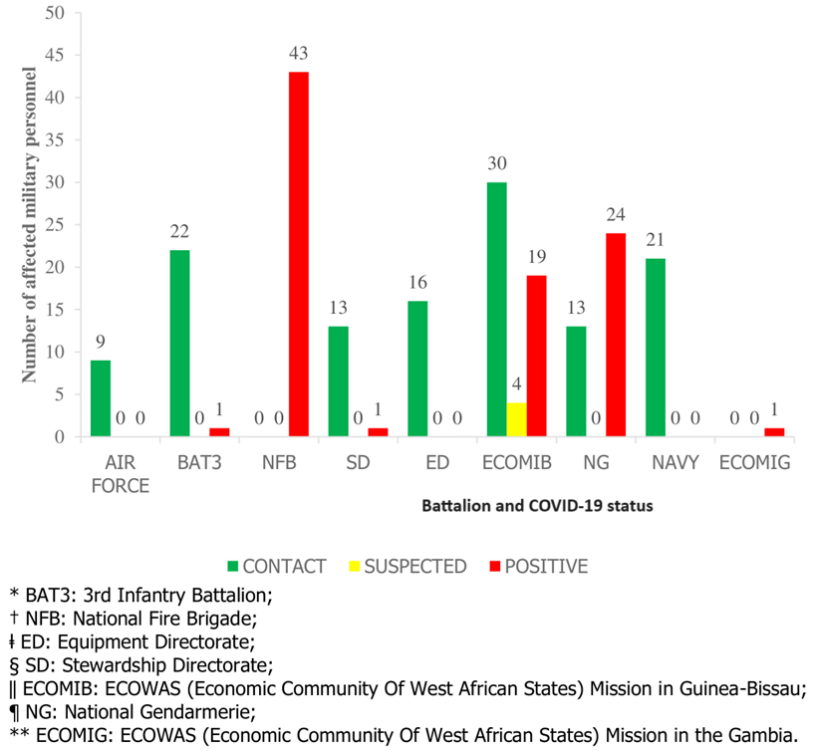
distribution of affected military personnel by battalion and COVID-19 status

**Study population:** our study included 217 Senegalese soldiers belonging to battalions of different military service branches of the armed forces (army, air force, navy, national gendarmerie, national fire brigade). One of the battalions was deployed overseas (Guinea Bissau) and another one was in pre-deployment for Gambia (Figure 1). The figure shows that the ECOMIB (ECOWAS (Economic Community of West African States) Mission in Guinea-Bissau), the NFB (National Fire Brigade), and the NG (National Gendarmerie) had most of the affected, with respectively 53, 43 and 37 soldiers. Our study population consisted of 58.1% non-commissioned members, 35.5% noncommissioned officers, and 6.4% officers. The sex ratio of males to females was 4.4. We did not include in the study 24 Stewardship Directorate (SD) soldiers deprived of their telephone during their qualification training, as well as one gendarmerie soldier and one NFB firefighter who declined the psychological support. In our study, 57.1% of cases were listed as contact, 41% as positive, and 1.9% as suspected. Figure 1 shows that the NFB had more positive cases and the ECOMIB had more contact cases.

**Figure 2:**
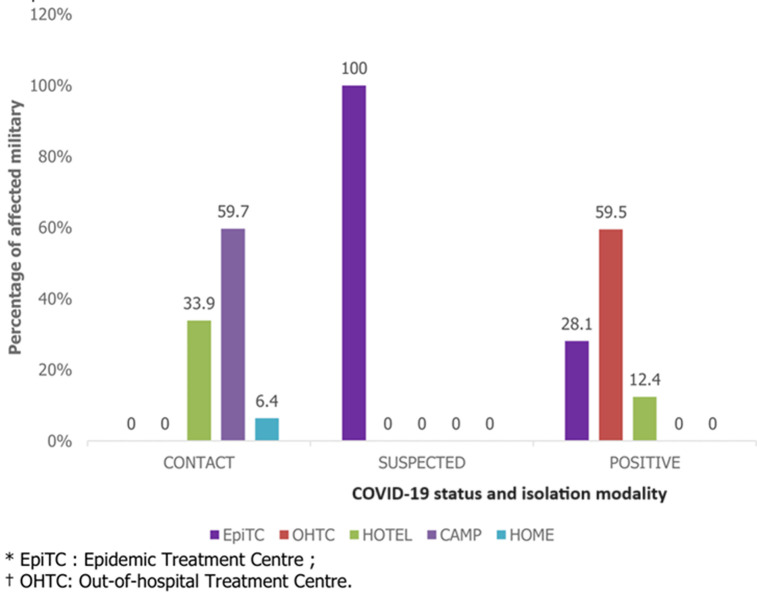
distribution of affected military by COVID-19 status and isolation modality

**Care management:** those affected by COVID-19 were taken care of in Epidemic Treatment Centres (EpiTC), Out-of-Hospital Treatment Centres (OHTC), hotel accommodations, or at home. In the case of the Military, military camps were used as isolation sites, as the epidemic progressed. Figure 2 shows that all suspected cases in our study were isolated in EpiTCs, while 59.7% of cases listed as contacts were isolated in military camps, and 59.5% of positive cases were managed in OHTCs. Those affected received psychological support for an average of 16 days, from April 12^th^ to July 20^th^, 2020, through telephone interviews of varying length and frequency depending on their condition.

**Stress and resilience factors:** the notes, collected during psychological support, have identified stress and resilience factors, categorized into health, sociological, and work-related factors. Table 1 lists the subcategories and codes for the stress and resilience factors.

**Table 1:** list of subcategories and codes for stressors and resilience factors

STRESS FACTORS	RESILIENCE FACTORS
**Health stress factors**	**Health resilience factors**
Care	Care
Isolation	Isolation
Experienced isolation	Experienced isolation
Isolation condition	Isolation condition
Diagnostic process	Diagnostic process
Relationship with medical team	-
Individual and disease	Individual and disease
**Sociological stress factors**	**Sociological resilience factors**
Family	Family
Family information	Information
Not informed	Support
Diverted information	-
Selective information	-
Disease and family	-
Risk of contamination	-
Member positivity	-
Awaiting results	-
Comorbidity	-
Family sociology	-
Apprehension	-
Remoteness	-
Management	-
Work environment	Work environment
Attitude of peers	Support of colleagues
Attitude of the hierarchy	Support from the hierarchy
General population	-
Stigma	-
Media coverage	-
**Work-related stress factors**	**Work-related resilience factors**
Training	-
Responsibilities	-
Sanction	-
Procedure	-

**Health-related stress and resilience factors:** health factors were related to care provided to those concerned and the individual´s relationship to disease. In terms of care management, isolation was experienced with loneliness, which was more acute for a female soldier and when the colleagues were released from the hospital before the others. This loneliness and the vacancy led to boredom, the cause of an inversion of a soldier´s sleep rhythm. The space was considered insufficient for quality sport. The isolation was felt as forced confinement with restricted movement. Some of the affected did not experience loneliness and vacancy thanks to the reunification of family members on the site and close, remote, or virtual connection with colleagues. The mutual support and bonding in the group contributed to a good atmosphere at the isolation site. They were able to stay busy with professional, domestic, sports activities (sometimes outside the site), leisure activities (internet), walks with even the possibility of going out at some of the sites. The isolation was considered long with a painful experience of uncertainty over its duration, but some were encouraged by the hope of a short isolation. Some soldiers were unable to make arrangements due to the lack of advance notice before the solitary confinement, resulting in the lack of cash and the difficulty in resolving needs on site. The isolation condition with the absence of certain items affected their comfort (television) or health security (hygiene kit). Food, in quantity and quality, was also a concern for the isolated. For others affected, the isolation in the usual housing or the workplace as well as the good conditions (environment and food), helped to avoid these inconveniences. In addition, the fact that the medical team provided good care conditions was helpful. During care, tests for the diagnostic process were a concern. This included uncertainty about the status of the disease with a long wait to be tested (initial or control test), an undetermined result, a negative result before being positive, and a painful sampling. In this area, it was reassuring for those affected to have an early test or negative income.

The lack of good communication between the patient and the medical team was also a concern. the military patient's relationship with the disease was characterized by several stress factors. Indeed, the fear of being positive for COVID-19 was present, linked to proximity to the positive cases despite the constant respect of protective measures, but also exposure in a professional context. This fear was heightened by the existence of an underlying somatic disease. On the other hand, others considered the possibility of contamination given the context and were reassured by compliance with the recommended protective measures or by distant contact with positive cases. The positivity of the initial or control test, which confirmed the disease, generated concern, which some defended by denial or rumination on the origin of the contamination. Some of those involved were able to cope by referring to God's will, previous experiences, a positive mental attitude, their solitary nature, or their status as medical personnel. The absence of disease symptoms or clinical improvement reassured some and others considered the disease as flu.

**Sociological stress and resilience factors:** the sociological stress and resilience factors encountered were related to the family and the work environment. In addition, stress factors related to society in general were identified. At the level of the family circle, the soldier's management of information on COVID-19 status could be a stress or resilience factor. Sharing information with the family has been complex. Some had chosen not to inform their families, others to find alibis to justify their absence (diverted information), and some to inform only a selected family member (selective information). For some, the option of informing the family, globally or selectively, allowed them to mobilize resources that helped during the isolation. Thus, the information shared enabled the family to stay in touch or to take into account certain family responsibilities of the person affected. The soldier was also affected by the possibility or reality of introducing the disease into their family (risk of contamination). For example, the fear of having infected a family member, especially young children's mothers, made it difficult to wait for the family's test results. The presence in the family of a member with a serious chronic disease (comorbidity) contributed even more. In terms of family sociology, remoteness was feared especially at the time of religious events. The distance made it difficult to manage the family, which had their organization disrupted during the isolation. The soldier had apprehensions about the reaction of his family to his return home.

In the work environment, the attitude of peers and hierarchy was, depending on the situation and the affected, a cause of suffering or in some cases a source of support. Colleagues stigmatized those affected by their attitudes (excessive caution) or sometimes by their joking remarks. This stigmatization generated guilt in situations of secondary contamination of colleagues, with apprehension about returning among them. In different situations, the apprehension about returning could be due to the uncertainty of the colleague´s status. The battalion´s colleagues supported the affected during the period of isolation by maintaining telephone or physical remote contact. They contributed to a good quality reintegration into the battalion after their treatment. In the relationship with the hierarchy, patients noted the non-supportive, even guilt-ridden attitude, or on the contrary, a hierarchy near to the involved, supportive, and attentive to their needs. Among the general population, stigma and media coverage of the soldier´s contamination were painfully experienced. Stigma by society was feared by the affected soldiers who apprehended the disclosure of their status. It resulted in the stigmatization of some battalions (ECOMIB contingent, Touba fire brigade) and hostile attitudes: refusal to continue housing them, demonstrations of the population against the establishment of a treatment center for them.

**Work-related stress and resilience factors:** the work-related stress and resilience factors identified were multifaceted. Ongoing training was interrupted while preparation for a professional examination and administrative procedures were disturbing. Similarly, isolation distanced them from their professional responsibilities and considerably reduced the battalion's strength to the point of becoming a concern for the operational capacity. The prospect of disciplinary action against some positive soldiers in their battalion was also resented by them. The professional anticipatory measures taken by some battalions helped to reassure soldiers. The prospect of resuming professional activities, or even of being deployed abroad, was a source of support during the isolation.

## Discussion

Our study aimed to investigate psychological issues in soldiers affected by COVID-19. Our study showed mainly that health, sociological, and occupational aspects were stress factors, but they generated resilience also.

**Methodology and study population:** the strength of our study lies in the fact that it focuses on a specific and singular population, military units. Studies on COVID-19 in the military environment exist but have focused more on the somatic and psychological complications much less on factors [3-5]. In this context, conducting the study during the first wave of COVID-19 enabled us to have an assessment of the early psychological aspects of the first encounter between the pandemic and the community [6]. Other strengths of the study are based on the clear framework of definition and classification of COVID-19 statuses and the interview methodology of the NCEM´s Psychosocial Commission. The first limits of the study are related to the temporal delimitation of the first wave and the sociological group concerned, subject to specific constraints. These aspects can influence the findings of the study and constitute a limit to the generalization of the conclusions to all military personnel or the general population. The selection bias of the study is because we included only concerned soldiers for whom the context of the request involved the AFPSP. The subjectivity of the practitioner and the patients, as well as the tools used, introduce a bias in the information measurement. The analysis as well as the qualitative and retrospective interpretation of interview notes also constitute a limit.

The size of our study population (n=217) seems small given the strength of the Senegalese armies estimated at 13,600 [7]. However, it includes units in each of the major entities of the armed forces, with even one deployed in external operations. All rank categories are represented in our series but in different proportions from the army´s establishment and staffing table (E.S.T.). The comparison shows that officers, non-commissioned officers, and women military are over-represented, and non-commissioned members are under-represented in the study population. Housing and working conditions should put non-commissioned members at greater risk for COVID-19. This distribution may be explained by the low presence of non-commissioned members in the national fire brigade and national gendarmerie units.

The Senegalese armed forces, in their various components, participated in the management of the pandemic by supporting the administrations in medical, security, and logistical terms. Some of its units were on the front line, with more affected elements as a result. In our study, this was the case for the contingent deployed in Guinea Bissau (ECOMIB) with a level 2 hospital at the service of the ECOWAS force and the population [8]. We also had a national fire brigade´s unit involved in the transport of patients in a pandemic epicenter. The national gendarmerie, involved in monitoring the implementation of governmental measures to fight the pandemic (curfew, state of emergency ...) was also on the front line. Statistical studies on COVID-19 in Senegal focused more on the accumulation of positive cases, as reported by Ndiaye [8] who counted 467 positive soldiers during the first wave. With a different methodology, in our series, the military listed as contacts were in the majority.

**Site of the care:** considering the contagiousness of the COVID-19 and its rapid progression in the world, countries, after declaring war on the disease, took restrictive and constraining measures. The distance was advocated and practiced between individuals, more towards the sick, suspects, or contacts. The principle of isolation was a major measure in the management of the disease [2]. The policy of isolation evolved considerably during the first wave in Senegal, due to the overload of the capacities put in place [9]. At the beginning of the pandemic, positives and suspects were managed in EpiTCs, while contacts were isolated in hotels. The overcrowding of the EpiTCs led to the establishment of out-of-hospital treatment centers (OHTCs) for the management of asymptomatic positives. This approach even evolved largely towards home self-isolation towards the end of the first wave [9]. Soldiers were included in the national management system. The care arrangements were therefore similar to those for the general population. The difference was the use of military cantonments as an isolation site as the epidemic progressed, and the additional psychological support provided by the AFPSP. For these reasons, we find in our study that more than the majority of contact and positive cases were isolated in the military cantonments and the OHTCs respectively.

### Isolation: a “solitary” confrontation between the soldier and the disease?

Our study showed that isolation, in a hospital or other structure, in unpreparedness and discomfort, without visibility over time, placing the individual in a boring loneliness, was the source of much inconvenience. In 2020, Brooks *et al*. [10], in a review of the literature on the psychological impact of quarantine, noted as stressors the boredom, the duration of quarantine, and the inadequacy of the means put in place. It was even argued that COVID-19 accelerated the vicious cycle that involved isolation, physical inactivity, and stress [11]. The soldier is characterized by his/her active and mobile status within a structured group. Isolation radically changes his/her environment by immobilizing him/her in a new environment and imposing an effort to adapt to constraints. Our study showed that isolation, under appropriate conditions, on a familiar site with or near familiar people, was beneficial. This seems to give a head start on coping and the individual can rely on the medical team as a substitute and move towards developing occupational strategies. The environment of isolation can improve the feeling of loneliness related to COVID-19 [6], and maintaining physical activity is of major interest to well-being [11]. Soldiers, subjected throughout their careers to changing and often hostile environments, develop coping skills. Thus, they may have fewer demands on the conditions of the host site and develop occupational activities and strategies for connecting with the rest of the group of colleagues.

The tests, in their execution and their results, were the cause of inconveniences that the quality of the communication with the medical team was often not able to resolve. In 2020, in his work, Brooks [10] emphasized that inadequate information from caregivers is a stress factor for people in quarantine. The perception of the disease during the first wave contributed to fear and anxiety about being “labeled” positive for COVID-19, generating defense mechanisms. This could evolve into psychological suffering as demonstrated by Minghuan Wang [12], in his study in China, among quarantined suspects and contacts. In addition to repositioning themselves concerning the perception of COVID-19, soldiers relied on their resilience that drew on their experience and character traits.

### Isolation: a sociological redefinition of relationships?

Isolation removes soldiers from their usual environments and has systemic, family, or institutional consequences. Our study reveals that family is a concern for the soldier. As in our study, the relevance of informing the family was raised by Desclaux *et al*. [13] during the follow-up of contacts of an Ebola virus patient in Senegal. The fear of contaminating the family as well as the absence of the soldier were present in our study. Military personnel are often absent from their families, especially during overseas deployments. These situations already distance them from their affective landmarks and their isolation keeps them away from colleagues who could be effective substitutes. We have found that if prepared, this family can be a reliable ally and a resource, in the management of this delicate period.

Our study showed that the situation could modify the relationship with colleagues, in the sense of stigma and development of a feeling of guilt. This stigmatization, which can arise from the fear of being contaminated, has been widely discussed in previous studies [10,13,14]. The cohesion between colleagues, a key strength of military units, has been put to the test by this stigma. The pandemic questioned the values of solidarity because it changed and redefined relationships between colleagues. Nevertheless, colleagues were an important asset in the well-being of the troops through the development of a network of “presence” with the isolated soldier. In 2017, Desclaux *et al*. [13], in their monitoring of Ebola contacts in Senegal, noted the role of the media in the development of stigma in society. The military felt singled out by the society they were supposed to support and protect. From media coverage to hostility, via stigmatization, the sense of honor and invulnerability of the (wo)man in uniform was eroded during this period in some units.

### Isolation, a professional brake?

The COVID-19 imposed a worldwide economic slowdown due to restrictive measures. The armed forces were not spared, with the isolation of the men putting a brake on the optimal functioning of the units. At the individual level, we have seen that it suspended or disrupted professional projects and led to administrative complications with military hierarchy.

## Conclusion

Assessment of the psychological aspects of the encounter between coronavirus disease and the military population, during isolation care in the first wave, revealed stress factors, but also resilience. These factors were related to health, sociological, and occupational aspects. Isolation suspends the plans of the soldier, removes him/her from his/her usual environment, and places him/her in an unusual environment or unfamiliar conditions, in front of his/her thoughts about the disease. Stigmatization also tests the cohesion within the unit and leads to rejection from society. In the face of these constraints, soldiers were able to rely on their personal coping resources and develop strategies. Colleagues and family members helped develop a “virtual presence” during the isolation. From this “solitary” confrontation with the disease, imposed by the isolation that redefined the environment, it appears that the Senegalese soldier, beyond his/her resources, was able to rely on his/her relatives and colleagues to alleviate the discomfort. Isolation, certainly a necessary measure to limit the spread of the contagious disease, has largely shown its psychological consequences, the foundations of which have been found in our study. In addition to relying on personal resources, the individual´s search for complete well-being then leads to rethinking the terms of isolation by also mobilizing actors of the immediate environment.

### What is known about this topic

- *The coronavirus disease (COVID-19) and the mechanisms put in place to control it have had a psychological impact on the population, especially those affected;*
- *Countries took restrictive and constraining measures; distance was advocated and practiced between individuals, more towards the sick, suspects, or contacts;*
- *The principle of isolation was a major measure in the management of the disease*.

### What this study adds

- *This study highlights psychological aspects of the encounter between coronavirus disease and the military population, during isolation care;*
- *This “solitary” confrontation with the disease revealed stress factors, but also resilience;*
- *In addition to relying on personal resources, the individual´s search for complete well-being then leads to rethinking the terms of isolation by also mobilizing actors of the immediate environment*.
